# Emergent Penile Venous Stripping for Treating Adolescent Impotence

**DOI:** 10.3390/life14060762

**Published:** 2024-06-14

**Authors:** Ko-Shih Chang, Yi-Kai Chang, Cho-Hsing Chung, Geng-Long Hsu, Jeff SC Chueh

**Affiliations:** 1 Division of Cardiovascular Medicine, Microsurgical Potency Reconstruction and Research Center, Yuan Rung Hospital, Yuanlin, Chenghua 51052, Taiwan; 2 School of Nursing, National Taipei University of Nursing and Health Science, Taipei 112303, Taiwan; 3 Department of Urology, National Taiwan University Hospital, Taipei 10002, Taiwan; 4 Department of Urology, Wan Fang Hospital, Taipei Medical University, Taipei 11696, Taiwan; 5 Microsurgical Potency Reconstruction and Research Center, Hsu’s Andrology and Shu-Tien Urology Ophthalmology Clinic, Taipei 10662, Taiwan

**Keywords:** adolescent erectile dysfunction, erection-related veins, penile venous stripping, psychogenic, venous leak, veno-occlusive dysfunction

## Abstract

Introduction: Traditional anatomy-based penile venous surgery is deemed inadequate. Based on revolutionary insights into penile vasculature, penile venous stripping (PVS) shows promise in treating adolescent erectile dysfunction (AED). We aimed to report on this novel approach. Methods: We conducted a retrospective analysis of 223 individuals under 30 diagnosed with veno-occlusive dysfunction (VOD) between 2009 and 2023. Among them, 83 were diagnosed with AED and divided into the PVS (*n* = 37) and no-surgery (NS, *n* = 46) groups. All participants had been dissatisfied with conventional therapeutic options. Dual pharmaco-cavernosography was the primary diagnostic modality. PVS involved stripping the deep dorsal vein and two cavernosal veins after securing each emissary’s vein with a 6-0 nylon suture. Erection restoration was accessed using the abridged five-item version of the International Index of Erectile Function (IIEF-5) score system and the erection hardness scale (EHS). Statistical analysis was performed using IBM SPSS 21.0. Results: There were significant differences (both *p* < 0.001) between the preoperative and postoperative IIEF-5 scores in the PVS and NS groups (9.8 ± 3.0 vs. 20.4 ± 2.2; 9.9 ± 2.5 vs. 9.5 ± 2.1), as well as in the EHS scores (1.7 ± 0.7 vs. 3.5 ± 0.6 and 1.8 ± 0.5 vs. 1.3 ± 0.4). The satisfaction rate was 87.9% (29/33) in the PVS group and 16.7% (17/41) in the NS group. Conclusions: AED can be effectively treated using physiological methods, although larger patient cohorts are needed for validation.

## 1. Introduction

In the medical literature, the earliest documented attempt to treat impotence in teenagers’ dates back to Porona’s venous injection of a hypertonic solution into a large vein for sclerosing penile veins in 1870, yielding questionable results [1]. However, this approach appeared to be rooted in conventional penile anatomy (CPA) [2]. Despite humanity’s existence for three millennia, the understanding of penile vascular anatomy was traditionally attributed to Sage Da Vinci before 1519. Consequently, CPA has prevailed in the medical literature for six centuries [3]. Prompted by inquiries from cardiologists and gynecologists, a revolutionary understanding of penile venous anatomy (RPVA) emerged in the 2000s [4]. Could this RPVA merit consideration as the final independent compartment in the human cardiovascular system, if venous drainage corresponds to arterial perfusion in each compartment of the human cardiac system? Could RPVA refine penile venous stripping (PVS), given that this updated understanding can guide PVS? Over the past two centuries, penile venous surgery, from its inception to refinement, has benefited erectile function across all age groups. But what about those experiencing adolescent-onset erectile dysfunction (AED)? [5,6,7,8].

Adolescence, typically spanning from ages 10 to 19, represents a phase of robust health for most individuals [9]. Regrettably, however, many adolescents experience premature mortality due to various causes [10]. Moreover, numerous serious adult-onset illnesses have their origins in adolescence. Psychogenic ailments often hinder adolescents from enjoying daily life and can detrimentally affect their academic performance, which is closely linked to emotional well-being [11]. While conventional medical wisdom attributes erectile dysfunction (ED) in men under 40 to psychogenic factors, our extensive clinical experience contradicts this notion. Over the years, we have consistently identified venous leaks or veno-occlusive dysfunction (VOD) as the predominant cause of ED in adolescents [12]. Through the application of penile venous stripping (PVS), we have effectively addressed the underlying physiological issues related to venous leaks in these individuals [13]. Our initial administration of PVS to five adolescents with erectile dysfunction dates to 1986. Since then, numerous adolescents have sought our assistance after receiving diagnoses of psychogenic ED from their primary care providers and failing to find resolutions through psychiatric referrals. In many of these cases, PVS alone has alleviated many issues stemming from ED. This therapeutic insight underscores the significance of VOD and its primary role in erectile dysfunction across all age groups.

The principle of personalized medicine is paramount in the management of any disease, including neurogenic erectile dysfunction (ED) [14]. It stands to reason that this principle should also apply to the treatment of psychogenic ED and veno-occlusive dysfunction (VOD). While modern medicine has made significant strides in the management of ED, perhaps none have been as groundbreaking as the introduction of phosphodiesterase-5 (PDE-5) inhibitors [15]. PDE-5 inhibitors are widely regarded as a panacea of ED of diverse etiologies [16]. However, it is essential to question the sustainability of this simplistic approach, as it fails to address critical issues encountered in clinical practice, such as why many adolescents with ED exhibit poor responsiveness to PDE-5 inhibitors [17].

In March 2009, a 24-year-old European boxer presented with AED and sought PVS despite his excellent physical health. He reported consistently insufficient penile rigidity and early detumescence before the age of 19. Despite previous diagnoses of psychogenic ED by other physicians, our evaluation, which included dual cavernosography and a prostaglandin-E1 test, confirmed a diagnosis of penile VOD [18]. The patient underwent successful ambulatory treatment with PVS, and we are grateful for his suggestion, which led to the grant of a patent by the United States Patent and Trade Office (USPTO) in 2012 [19]. Similarly, in May of the same year, a concerned father traveled across the Pacific Ocean with his 25-year-old son seeking PVS. His son had experienced markedly poor erection quality since the age of 16, leading to concerns about academic performance. Despite consultations with five renowned urology professors, all of whom attributed the ED to psychological issues, the patient’s condition continued to deteriorate socially and academically. Fortunately, PVS successfully resolved his refractory AED. Cases like these are not uncommon. For example, in July 2015, the mother of a junior medical student at a prestigious university sought our assistance. Her son, despite being academically driven, had been severely affected by AED since the age of 17, leading to an inability to continue his studies. Despite consultation with accredited urologists who attributed the ED to psychogenic causes, psychiatric management failed to address the primary concern. Dual cavernosography confirmed the presence of penile VOD, highlighting its significant role in AED without comorbidities, akin to those whose ED resulted from cigarette smoking [20].

Alongside our chronological practices, we observed that PVS effectively treated AED in patients, often obviating the need for psychiatric management and facilitating a return to scholastic achievement within several months postoperatively. Inspired by these cases involving international AED patients, we embarked on a retrospective analysis of ambulatory PVS as a treatment modality for AED. This endeavor aimed to address the existing gap in medical research concerning this specific patient population.

## 2. Methods

### Patient Population

After receiving approval from the institutional review board of China Medical University, we conducted a retrospective study spanning from March 2009 to May 2023. During this period, a total of 223 patients, all under the age of 30, underwent PVS for the treatment of VOD, despite each having previously been diagnosed with psychological causes of ED elsewhere internationally. This diagnosis of psychogenic ED was made after a comprehensive multidisciplinary investigation failed to identify any organic causes of ED. Patients were excluded from the study if their ED occurred after the age of 19 and included if their ED began before the age of 19. Among them, 46 men declined PVS despite documented VOD, and PVS was recommended at our institute. Consequently, 83 AED patients were allocated into two groups: the PVS group (*n* = 37) and the no-surgery (NS) group (*n* = 46), where men with AED were under observation without intervention but were prescribed oral medication as required for control. Within the PVS group, two patients had been diagnosed with schizophrenia at the ages of 13 and 14, respectively, and were under acceptable control.

**Figure 1:**
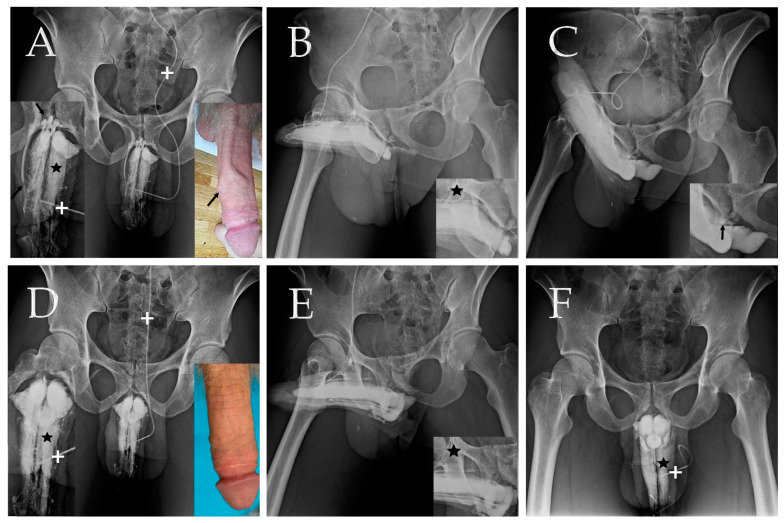
Dual pharmaco-cavernosography of a 27-year-old international patient with AED. (**A**) The first cavernosogram (anterior–posterior view) captured during the injection of 10 mL diluted iohexol solution into the corpora cavernosa (asterisk, inserted left) via a no. 19 scalp needle (cross, inserted left). Notably, the superficial dorsal vein (arrow inserted left and right with a preoperative self-injecting cavernosogram) exhibits a direct shunt with the corpora cavernosa (CC). The pre-prostatic venous plexus (curve arrow, inserted left) becomes immediately visible due to the pronounced drainage of the CC. (**B**) A 30° oblique-view cavernosogram acquired after an additional injection of 10 mL iohexol solution. The substantial superficial dorsal vein extends along the penile shaft and maintains communication with the CC up to the penile base (asterisk inserted). (**C**) This pharmaco-cavernosogram illustrates a venous channel despite the induction of an artificial erection via intracavernous injection of 20 μg prostaglandin E1, confirming the presence of VOD (arrow, inserted). (**D**) The imaging procedure replicated from panel (**A**); this postoperative cavernosogram was taken after penile venous stripping. The superficial dorsal vein is no longer visible (inserted). Notably, the CC demonstrates radio opacity (asterisk). One year postoperatively, using a no. 19 scalp needle (cross), a self-injecting cavernosogram was performed, revealing the absence of offensive veins (inserted right), providing a good comparison with panel (**A**). (**E**) An oblique cavernosogram obtained for comparison with panel (**B**). The substantial venous plexus is no longer observed (asterisk inserted). The corpus spongiosum becomes a prominent route of venous drainage. (**F**) A postoperatve cavernosogram, using a no. 19 scalp needle (cross) demonstrated significantly enhanced intracorporeal fluid retention of CC (asterisk).

**Figure 2:**
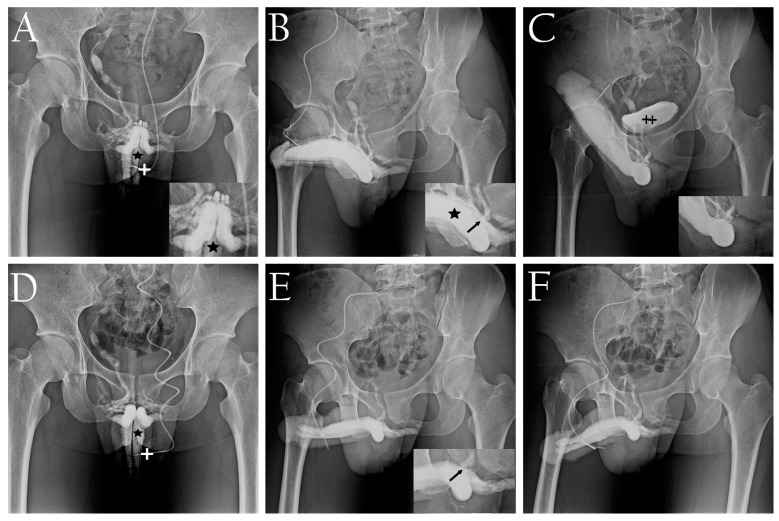
Dual pharmaco-cavernosography of a 20-year-old medical student in the adolescent group. (**A**) The first cavernosogram (anterior–posterior view) acquired during the injection of 10 mL diluted iohexol solution into the corpora cavernosa (asterisk) via a no. 19 scalp needle (cross). Multiple channels of the pre-prostatic venous plexus are evident. (**B**) A 30° oblique-view cavernosogram obtained after an additional injection of 10 mL iohexol solution. Note the antler-like appearance of the penile erection veins (arrow and asterisk inserted). (**C**) This pharmaco-cavernosogram confirms veno-occlusive dysfunction (inserted) despite the induction of an artificial erection by intracavernous injection of 20 μg prostaglandin E1. The antler-like appearances of the penile erection-related veins persist, albeit slightly reduced in size. The urinary bladder (double cross) is well demonstrated. (**D**) Using a no. 19 scalp needle (cross), an anterior–posterior view cavernosogram for comparison with panel (**A**), revealing scattered erection-related veins. The superficial dorsal vein is no longer visible (inserted). The erection-related veins are absent, consequently the intracorporeal fluid retention of CC (asterisk) is enhanced. (**E**) A 30° oblique-view cavernosogram for comparison with panel (**B**) after penile venous stripping. Postoperatively, the erection-related veins are no longer visible (inserted), and the imaging of the penile drainage veins within the pelvis is significantly diminished (arrow, inserted). (**F**) A 30° oblique-view cavernosogram, using a no. 19 scalp needle demonstrated significantly enhanced intracorporeal fluid retention of CC.

The primary diagnosis tool utilized in this study was our dual cavernosography technique. In this procedure, the first set of cavernosograms (depicted in Figure 1A,B) was obtained by injecting 20 μg of prostaglandin E1 (PGE-1) intracavernously using a no. 19 scalp needle. It is worth noting that the needle was inserted briskly with the bevel parallel to the dorsal artery to avoid traversing the artery. The cavernous artery flow was measured during this injection. Subsequently, a second set of pharmaco-cavernosograms (illustrated in Figure 1C and Figure 2C) was obtained through the same injection line. These images aided in the diagnosis of VOD [18]. Following the surgical procedure, the first dual cavernosography set was routinely performed postoperatively to confirm the thorough stripping of the offending erection-related veins (depicted in Figure 1D–F). This imaging protocol was exemplified in the PVS group (Figure 2).

**Figure 3:**
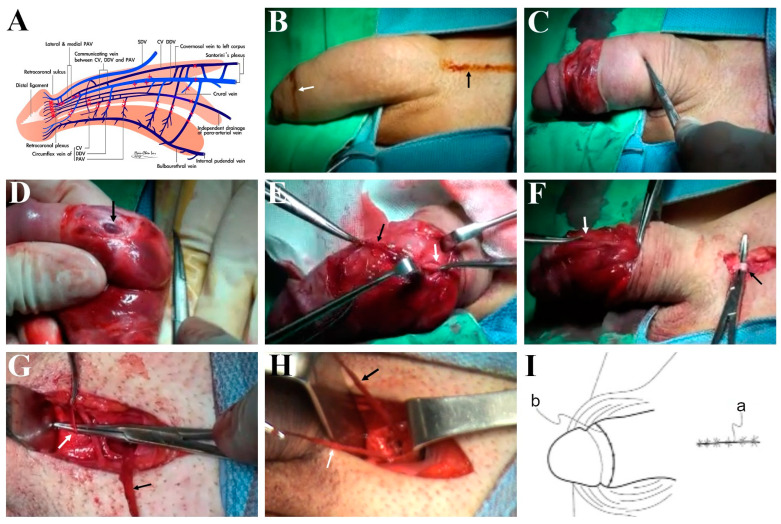
Schematic illustration of the innovative penile anatomy leveraged in this study and ongoing penile venous stripping procedure on a 24-year-old patient in the adolescent group. (**A**) From a lateral perspective, the erection-related veins include a deep dorsal vein (DDV), consistently in the median position, receiving blood from the emissary veins in the corpora cavernosa and the circumflex vein in the corpus spongiosum. The cavernosal veins (CVs) are specific to their corresponding corpus, lying deep. Each dorsal artery is flanked by medial and lateral para-arterial veins (PAVs). The internal pudendal artery branches to the bulbourethral artery and subsequently to the cavernosal artery, the major supplier to the CC, and the dorsal artery, supplying blood to the glans penis. (**B**) The procedure utilizes circumferential (white arrow) and pubic longitudinal (black arrow) approaches. (**C**) Circumcision is initially performed to access the DDV, which has a digital distribution. (**D**) Visibility of the DDV (arrow) can be enhanced through milking manipulation, akin to squeezing a balloon, on the corpora cavernosa. (**E**) The confluent trunk of the DDV (black arrow) is meticulously managed while all emissary veins are ligated to prevent bold gushing from the cavernosal sinusoids. The stripping procedure is step-by-step via a pull-through maneuver (white arrow), with the DDV confluent trunk serving as a guide. (**F**) Five pull-through maneuvers are typically required to reach the penile base. Subsequently, the same stripping procedure is applied to the CV system, with the PAVs subjected to segmental ligation. After making a 3 cm pubic longitudinal wound, the confluent trunk of the DDV and CVs (white arrow) is passed underneath the pubic wound (mounted by a hemostat, black arrow). (**G**) The stripping continues, with 15 venous branches to be ligated in the DDV system (black arrow) and then the CV system (white arrow) until reaching the infrapubic angle. (**H**) At least two substantial venous trunks are visible (white and black arrow). (**I**) Finally, both wounds are closed with 5-0 chromic or 6-0 nylon sutures.

Informed by the revolutionary erection-related venous anatomy (Figure 3A) and guided by the preoperative venous anatomy, the procedure was conducted under acupuncture-aided local anesthesia (AALA) on an ambulatory basis [21]. First, a circumferential approach was marked (Figure 3B) and executed, along with circumcision, if necessary (Figure 3C), followed by de-gloving to expose Buck’s fascia. The deep dorsal vein (DDV) system (Figure 3D) was identified, with its visibility enhanced by manual milking manipulation of the corpora cavernosa. Subsequently, it was stripped distally at the retro-coronal plexus, with 29–33 ligatures to confine the sinusoidal blood from the glans penis. This stripping process continued proximally along the penile shaft to the base, with each emissary ligated firmly closest to the outer tunica layer via a pull-through maneuver (Figure 3E). Similarly, the paired cavernosal veins (CVs) were managed, followed by segmental ligation of the two-pair para-arterial veins (PAVs). Milking manipulation was used to enhance visibility for deeper-seated residual veins. The DDV and CV venous network served as a guide for further proximal venous stripping. Subsequently, a 3 cm longitudinal pubic wound was made to facilitate penile venous stripping, with the venous stumps passed underneath it (Figure 3F). The DDV and CV systems were managed with 7–9 and 6–8 branches, respectively, up to the infrapubic angle, where at least two substantial venous trunks were apparent (Figure 3G). During the procedure, neither electro-cautery nor a suction apparatus was employed. A total of 132 ligations were required. Finally, both wounds were sutured with 5-0 chromic or 6-0 nylon sutures (Figure 3I).

**Figure 4:**
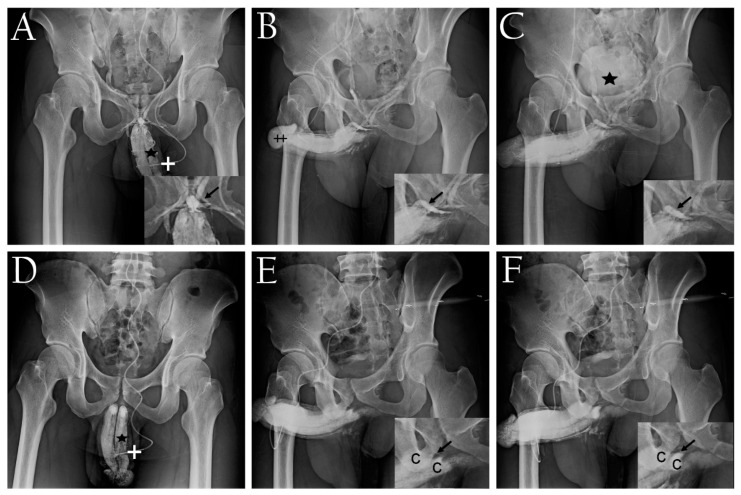
Dual pharmaco-cavernosography of a 25-year-old domestic patient with AED. (**A**) The first cavernosogram (anterior–posterior view) was obtained with a 10 mL diluted iohexol solution injected into the corpora cavernosa via a no. 19 scalp needle (white cross). Note the pooling of erection-related venous blood in the proximal deep dorsal vein (black arrow inserted right). The pre-prostatic venous plexus is prominently visible due to the pronounced drainage of the corpora cavernosa (black asterisk), extending even to the inferior vena cava. (**B**) A 30° oblique-view cavernosogram was captured after an additional injection of 10 mL iohexol solution. The extensive venous drainage reaches the inferior vena cava, along with the glans penis (black double cross) and the vast proximal deep dorsal vein (inserted right, black arrow). (**C**) Despite an artificial erection induced by an extracavernous injection of 20 μg prostaglandin E1, this pharmaco-cavernosogram reveals a venous channel, documenting veno-occlusive dysfunction (black arrow, inserted right), despite the penile erection achieving an intracorporeal pressure over 110 mmHg, with the urinary bladder shown (black asterisk). (**D**) This postoperative cavernosogram, duplicated from panel (**A**) at the patient’s request, via a no. 19 scalp needle (white cross), was obtained two years postoperatively. The deep dorsal vein is no longer visible, and the radio-opacity of the corpora cavernosa (black asterisk) is enhanced (black asterisk)—a notable improvement compared to panel (**A**). (**E**) An oblique cavernosogram obtained for comparison with panel (**B**), showing minimal evidence of the substantial venous plexus (black arrow inserted lower right). Note the visualization of the penile crura (C and C inserted right lower), with little venous drainage evident within the pelvic cavity. (**F**) Subsequent cavernsogram revealing enhanced intracorporeal fluid retention, with the erection-related veins stripped out (arrow indicates the position of erection-related veins preoperatively, inserted right lower) and the penile crura strongly opacified, confirming intracorporeal retention (C and C inserted right lower) parallel with remarkable erection restoration.

Meanwhile, a man with AED began experiencing this debilitating condition at the age of 13. Despite trying various contemporary ED therapeutic strategies, including oriental medicine, PVS was eventually conducted after confirming penile erection-related venous anatomy (Figure 4A,B) and VOD (Figure 4C). This well-educated individual later became a father and, two years postoperatively, requested imaging for comparison. Postoperative films demonstrated minimal venous imaging within the pelvis (Figure 4D–F). Overall, the IIEF-5 score system and radio-opacity were utilized to assess erectile function restoration, with the erection hardness scale (EHS) serving as a supplemental tool to confirm improvements in erectile rigidity.

In each patient, a radio-opacity index was utilized to compare the preoperative and postoperative radio-opacity of the penile crus versus the femoral cortex (Figure 1B vs. Figure 1E; Figure 2B vs. Figure 2E). Postoperatively, the IIEF-5 score, EHS, and CSI (Couples’ Satisfaction Index), albeit incomplete, were obtained through various means such as in-person interviews, telephone calls, or communication via public platforms like the Internet, Line, WeChat, etc. Data are presented as means (±SD) with the range, and statistical analysis was performed using IBM SPSS Statistics 21.0.

## 3. Results

The longest follow-up time from PVS in this study is 15.0 years, with a range between 0.8 and 15.0 years. The duration of the operation ranged from 4.1 to 6.5 h, with minimal blood loss. Table 1 provides a summary of the general data of the 83 patients who underwent PVS for treating AED in men, offering a comprehensive overview. Table 2 presents the demographic distribution of the 37 AED patients, all from financially secure families. Among them, 23 patients had the habit of masturbating twice per day until the ages of 14 (*n* = 11), 15 (*n* = 8), and 16 (*n* = 4) before experiencing poor erection rigidity. All AED patients experienced depressive episodes, with 78.4% (29/37) having suicidal ideation and 73.0% (27/37) undergoing psychiatric management, which, while beneficial for their psychological well-being, did not address their AED. Three patients had received treatment for schizophrenia for a decade.

There was a significant difference (Both *p* < 0.001) between the preoperative IIEF-5 scores (9.8 ± 3.0 vs. 20.4 ± 2.2; 9.9 ± 2.5 vs. 9.5 ± 2.1) and EHS scores (1.7 ± 0.7 vs. 3.5 ± 0.6; 1.8 ± 0.5 vs. 1.3 ± 0.4) for the PVS and NS groups, respectively. All 37 patients exhibited excessive penile venous vasculature (Figure 1A,B and Figure 2A,B), and VOD was categorically documented and diagnosed (Figure 1C and Figure 2C) via pharmaco-cavernosography. The preoperative radio-opacity of the penile crus was inconspicuous relative to that of the femoral cortex (Figure 1A–C and Figure 2A–C) but was enhanced postoperatively (Figure 1D–F and Figure 2D–F). All patients reported improvements in the EHS.

Once the PVS of the erection-related veins has been performed, the corpus spongiosum and the superficial dorsal vein become important circulation routes (Figure 1E and Figure 2E). A 22-year-old senior undergraduate sustained a firm erection for eight hours on the first postoperative morning; it was relieved by defecation before we planned for priapism management. The sleep pattern of several patients was ruined by waking up because of painful nocturnal erections for 2–3 weeks unexceptionally. In the PVS surgery perse, we encountered neither penile numbness nor penile deformity; thus, there were no surgery-related complications, intraoperatively and postoperatively. The postoperative penile shortage was temporary, and penile length returned to normal within four months postoperatively. However, during the diagnostic work-up, a severe allergy developed in one 27-year-old man, manifested as nausea, vomiting, flushing, lip and eyelid edema, and generalized urticaria during dual cavernosography.

Surprisingly, complete satisfaction was reported in those two males with AED who underwent psychiatric management in a schizophrenic special clinic, not only in scholastic achievement but also in couples’ harmony. Overall, the rate of sexual life satisfaction was 87.9% (29/33) in AVD patients and 16.7% (17/41) in the PVS and NS groups, respectively. In the PVS group, 18.9% (7/37) of patients sought the additional assistance of PDE-5 inhibitors, although the dosage was one-quarter to one-half. This dosage contrasted the mild or null response to this agent preoperatively, and one patient occasionally resorted to the penile intracavernosal injection of prostaglandin E1. Unfortunately, some patients were eventually excluded due to the inconsistent feedback of follow-up; this was the case for four and five men with AED in the PVS and NS groups, respectively. This included one AED patient who underwent malleable penile implantation elsewhere under strong request after a sexual partner’s insistence five years after PVS in 2015.

## 4. Discussion

In medical history, medical therapy typically takes precedence over surgical intervention, and the same holds true for adolescent erectile dysfunction (AED). In the age of oral medications for erectile dysfunction (ED), physicians must explore various surgical options for AED when conventional medical therapy fails. For instance, in the context of restoring male erectile function, it remains uncertain whether a surgical intervention can benefit individuals afflicted with ED. Given the limitations of conventional penile anatomy (CPA), should a traditional approach to penile venous surgery (CVPVS) be considered feasible based on CPA? [22,23]. CVPVS has been a subject of controversy since its inception in 1895, often viewed as experimental [24]. While some proponents argue for its efficacy [25,26], its merits have been debated for over three centuries. It has gained notoriety for causing irreversible penile deformities and untreatable penile numbness [27], often attributed to the use of electrocautery and inadequate venous removal. Consequently, urological guidelines do not recommend penile vascular treatments [28]. As a result, the focus has shifted towards understanding the pathophysiology of ED, particularly the role of psychogenic factors [29,30]. Anatomy and physiology are rudimentary for every therapeutic strategy [31]; however, should CVPVS be exempt from rigorous anatomical and physiological considerations? This question warrants thorough discussion, especially since the prototype of penile venous stripping (PVS) was introduced in 1986 [13]. Human sexual health hinges on both psychological and physical well-being, with ED arising when either aspect is compromised [32]. Recent studies have highlighted the pivotal role of penile erection-related veins in determining erection rigidity on defrosted human cadaveric penises [33]. Should venous occlusive dysfunction (VOD) be reconsidered if psychogenic factors are deemed irrelevant in the erection process? [34]. Consequently, the placebo effect is eliminated. Adolescents can achieve rigid erections through various stimuli, suggesting a psychogenic component. However, should VOD still be considered a contributing factor? These questions underscore the need for a comprehensive understanding of the underlying mechanisms of AED and the potential role of surgical interventions in its management.

Certainly, considering the significant association between adolescent erectile dysfunction (AED) and venous occlusive dysfunction (VOD), it seems appropriate to re-evaluate the classification of contributors to erectile dysfunction (ED). Including VOD alongside psychogenic factors, hormonal imbalances, arterial issues, neurogenic causes, adverse drug effects, and systemic diseases could provide a more comprehensive framework for understanding and addressing AED. The proposed acronym VPHANDS reflects the diverse etiological factors involved, with each contributing to the complex interplay that leads to ED in adolescents. Given that masturbation is a common experience among AED individuals, recognizing the role of VOD in this context becomes crucial. Failure to consider VOD as a potential cause may lead to challenges in explaining why AED patients are unresponsive to conventional treatments like PDE-5 inhibitors. Thus, expanding the ED contributors list to include VOD could enhance diagnostic accuracy and guide more tailored treatment approaches for AED [35,36].

Given that organic ED is a predictor of increased future morbidity and mortality, a thorough check-up is recommended for any man complaining of sexual dysfunction [37]. In AED, imminent morbidity and mortality are possible due to suicidal ideation. A multidisciplinary investigation might be necessary to rule out the organic factor regardless of marked lower probability. Investigation tools entail a detailed physical examination, a routine biochemistry profile, an endocrinology assay, nocturnal penile tumescence and rigidity recording, penile vascular investigation, and neurotic and psychiatric tests. Some studies have suggested that children and adolescents are more prone to respond to a placebo than adults.

Furthermore, a man with AED at age 25 reported a rapid recovery after PVS supplemented with psychotherapy in the advanced North European country; we believe that psychogenic tests should be exclusively mandatory in AEDs. Androgens play a paramount role in sexual function, particularly in those whose age reaches male menopause (accompanied by testosterone deficiency); ED is common in this case [38]. AED diagnoses are most accessible from this concern; we encountered a 27-year-old man with AED who underwent a pituitary adenectomy followed by periodic hormone replacement, and PVS upgraded his IIEf-5 from a score of 17/25 to 25/25 in just 6 weeks after ambulatory PVS. Due to this study’s paucity of similar cases, we will no longer discuss the endocrinologic relevance. Our success was built upon our RPVA, which directed us to the chronological refinement of PVS. In addition, we thank acupuncture-assisted local anesthesia, which materialized the ambulatory basis in 1988. An ongoing PVS patient is conscious and immutable, which is beneficial for identifying muscle twitching if the innervated branch of the anterior muscle group of the bulbospongiosus muscle is inadvertently ligated, as was the case in 1999. From our vast experience of having performed many dozen salvaging PVS procedures on those who underwent CVPVS elsewhere internationally [39], we conducted repeat analysis, and two leading causes were identified as unsuccessful. First, the attending surgeon may fail to decline the temptation of applying electrocautery during procedures, causing a catastrophic thermal effect on the delicate corporeal sinusoids. Secondly, further scientific research is warranted to determine the effect of the incomplete removal of the offending erection-related veins for VOD.

Penile sinusoids are super A1 blood-nourished independent compartments located in the entire cardiovascular system, including the glans penis, the corpus spongiosum, and the corpora cavernosa, where cotton soft and bony rigidity are reciprocated by 2–3 to 60–80 mL/Min arterial perfusion. In other words, penile blood accounts for 2.5–3.2% of cardiac output in sexual arousal, which is a soaring increase of 30–40 times compared with the 1.79 times increase in the lower leg arterial perfusion rate from rest to running [38]. This implies that penile erectile function requires a functional heart. Despite AED’s gratifying outcome encouraging our continuation in this eccentric practice, in the era of oral therapy for ED solution, evidence can speak volumes and practice is the exclusive criterion for testing the truth, so medical practice stands. At present, our proof is just reaching level 3. By adopting the vast benefits of heavy health and nursing care costs of CABG surgery for endovascular coronary arterial stenting [40], we could aim to share and reproduce young surgeons. PVS’s ambulatory strategy can lower the cost burden of CVPVS, which adds up due to unsuccessful cases and health and nursing care costs [41]. We apply a randomized, double-blind, controlled study on refined PVS to provide level 1 evidence.

## 5. Conclusions

In conclusion, adolescent erectile dysfunction (AED) may not solely stem from psychological factors, as penile erection-related veins can play a significant role in its development. While AED is treatable, it remains a challenge to prevent.

## Figures and Tables

**Table 1:** Summary of the 83 patients whose impotence incepted before age 19 and received penile venous stripping from 2009 to 2023.

Group	Patient		Masturbation		IIEF-5 ^a^ Score			Additional Therapy		
	No.	Age (Year)	Experience	ED Related	Preop	Postop (6M)	Postop (1Y)	PDE-5i ^b^	ICI ^c^	Implant
PVS	37	23.1 ± 3.1	33	29	9.8 ± 3.0	18.2 ± 4.2	20.4 ± 2.2	2	1	1 ^d^
Control	46	23.2 ± 3.0	41	83	9.9 ± 3.5	17.9 ± 4.3	20.6 ± 2.3	9	7	0
Total	173	31.2 ± 3.1	153	106	9.9 ± 3.6	18.1 ± 4.2	20.5 ± 2.5	11	8	1
*p*-value ^e^	<0.01	NA ^f^	NA	NA	NA	both < 0.01 ^g^	both < 0.001	NS ^h^	NS	NS

^a^ IIEF-5 indicates the abridged 5-item version of the International Index of Erectile Function score system. ^b^ PDE-5i is an abbreviation for phosphodiesterase type 5 inhibitor. ^c^ ICI denotes intracavernosal injection of prostaglandin-E1. ^d^ One 26-year-old patient from South America received penile implant 4 years after undergoing penile venous stripping because of pelvic trauma. ^e^ A statistically paired *t*-test was used in those patients whose postoperative period was longer than 6 months. ^f^ NA stands for not applicable. ^g^ Data are calculated from the preoperative and postoperative data for each respective group. ^h^ NS stands for not significant.

**Table 2:** Demography of 37 patients whose ED onset at their adolescence in this study.

	Patient				Suicidal Thoughts	IIEF-5 ^a^ Score		Additional Therapy		
Native	No.	Age (Year)	STs ^b^	PM ^c^	Preop	Postop	Postop	PDE5i ^d^	ICI ^e^	Implant
						(6M)	(1Y)			
America	9	24.3 ± 3.5	5	4	9.5 ± 2.9	13.8 ± 4.4	20.6 ± 2.1	1	0	1 ^f^
Europe	8	24.1 ± 3.3	5	4	9.6 ± 3.1	13.8 ± 4.4	20.6 ± 2.1	0	0	0
AP ^g^	19	25.2 ± 3.4	17	15	9.7 ± 3.2	13.8 ± 4.4	20.6 ± 2.1	2	1	0
Africa	1	23	0	0	9	22	23	0	0	0
Total	37	25.1 ± 3.1			9.6 ± 3.2	20.6 ± 2.1	20.6 ± 2.1	16	4	10
*p*-value ^h^	NA ^i^	NA	27	23	NA	both < 0.0 ^j^	both < 0.0001	NS ^k^	NS	NS

^a^ IIEF-5 indicates the abridged 5-item version of the International Index of Erectile Function score system. ^b^ STs is an abbreviation of suicidal thoughts. ^c^ PM is an abbreviation of psychiatrist management. ^d^ PDE-5i stands for the phosphodiesterase type 5 inhibitor. ^e^ ICI denotes intravenous injection of the prostaglandin-E1. ^f^ One 26-year-old patient from South America underwent a penile implant 4 years after penile venous stripping because of pelvic trauma. ^g^ AP denotes the Asia–Pacific region. ^h^ Statistically paired *t*-test was used in those whose postoperative period was longer than 6 months. ^i^ NA stands for not applicable. ^j^ Data are calculated from the preoperative and postoperative data for the respective group. ^k^ NS stands for not significant.

## Data Availability

The data for this research were drawn from extensive clinical records spanning decades and were anonymized to protect patient privacy.
